# Diffractometer for element-specific analysis on local structures using a combination of X-ray fluorescence holography and anomalous X-ray scattering

**DOI:** 10.1107/S1600577524011366

**Published:** 2025-01-01

**Authors:** Hiroo Tajiri, Shinji Kohara, Koji Kimura, Sekhar Halubai, Haruto Morimoto, Naohisa Happo, Jens R. Stellhorn, Yohei Onodera, Xvsheng Qiao, Daisuke Urushihara, Peidong Hu, Toru Wakihara, Toyohiko Kinoshita, Koichi Hayashi

**Affiliations:** a Japan Synchrotron Radiation Research Institute Hyogo679-5198 Japan; b Center for Basic Research on Materials National Institute for Materials Science Ibaraki305-0047 Japan; c Department of Physical Science and Engineering Nagoya Institute of Technology Nagoya466-8555 Japan; d Graduate School of Information Sciences Hiroshima City University Hiroshima731-3194 Japan; e Co-Creation Institute for Advanced Materials Shimane University Matsue690-8504 Japan; f Institute for Integrated Radiation and Nuclear Science Kyoto University Osaka590-0494 Japan; g State Key Laboratory of Silicon Materials & School of Materials Science and Engineering Zhejiang University Hangzhou310027 China; h Division of Advanced Ceramics Nagoya Institute of Technology Nagoya466-8555 Japan; i Institute of Engineering Innovation, School of Engineering The University of Tokyo Tokyo113-8656 Japan; ESRF – The European Synchrotron, France

**Keywords:** X-ray diffractometers, X-ray fluorescence holography, anomalous X-ray scattering, element-specific measurements, carry-in equipment

## Abstract

To tackle disorder in crystals and short- and intermediate-range order in amorphous materials, such as glass, we developed a carry-in diffractometer to utilise X-ray fluorescence holography (XFH) and anomalous X-ray scattering (AXS), facilitating element-specific analyses with atomic resolution using the wavelength tunability of a synchrotron X-ray source. Our diffractometer unifies XFH and AXS configurations to determine the crystal orientation via diffractometry. In particular, XFH was realised even for a crystal with blurred emission lines by a standing wave in a hologram, and high-throughput AXS with sufficient count statistics and energy resolution was achieved using three multi-array detectors with crystal analysers. These features increase tractable targets by XFH and AXS, which have novel functionalities.

## Abstract 2

To tackle disorder in crystals as well as short- and intermediate-range order in amorphous materials, and increase tractable targets with novel functionalities, we have developed a carry-in diffractometer to utilise X-ray fluorescence holography and anomalous X-ray scattering, facilitating element-specific analyses with atomic resolution using the wavelength tunability of a synchrotron X-ray source.

## 1. Introduction

Wavelength tunability, a key advantage of synchrotron radiation, facilitates element-specific analysis of structures in materials. Several analytical methods, such as X-ray absorption fine structure, photo-electron spectroscopy, resonant X-ray scattering, and multi-wavelength anomalous diffraction in protein crystallography, capitalise on this advantage. The progress in the brilliance of synchrotron radiation (Yabashi & Tanaka, 2017 ▸) has accelerated the application of wavelength tunability for a wide variety of characterisation techniques, even in the field of diffractometry, whereas anomalous terms in X-ray atomic form factor realise element-specific analysis. Meanwhile, the demand for the characterisation of local disorder or order at the atomic level in materials science is increasing, assisted by the ongoing rapid progress in nanotechnology. This is because such local disorder or order represented by environmental structures around a dopant in a semiconductor or both short- and intermediate-range order in glasses plays a crucial role in the characteristics of materials, even triggering the emergence of new functionalities.

In fact, although the means to access such local structures are limited, not only a well known interstitial or substitutional structure by a dopant but also nanoclusters formed by dopants, slight distortions in relaxor ferroelectrics with nanoscopic heterogeneity (Hayashi & Korecki, 2018 ▸), dopant–vacancy pairs (Nagaoka *et al.*, 2023 ▸), large displacement of a dopant (Yamamoto *et al.*, 2022 ▸), a two-dimensional van der Waals material (Eguchi *et al.*, 2024 ▸), and even soft matter including protein crystals (Ang *et al.*, 2023 ▸) have become tractable by X-ray fluorescence holography (XFH).

Moreover, while understanding ‘order within disorder’ (Salmon, 2002 ▸) beyond the nearest-neighbour distance in disordered materials (*e.g.* liquid and glassy materials) remains challenging owing to the absence of translational periodicity and complexity in their structures, quantum-beam diffraction combined with advanced topological analysis has been addressing this (Salmon *et al.*, 2005 ▸; Kohara & Salmon, 2016 ▸).

To access these unique structures (*i.e.* disorder in order and order within disorder) and increase tractable targets with novel functionalities, we developed a carry-in diffractometer that utilises XFH and anomalous X-ray scattering (AXS), facilitating element-specific analyses with atomic resolution. Our diffractometer realises XFH even for a crystal with blurred emission lines by a standing wave in a hologram and high-throughput AXS with sufficient count statistics and energy resolution using three multi-array detectors with crystal analysers.

## 2. Materials and methods

### 2.1. Overview

**Figure 1:**
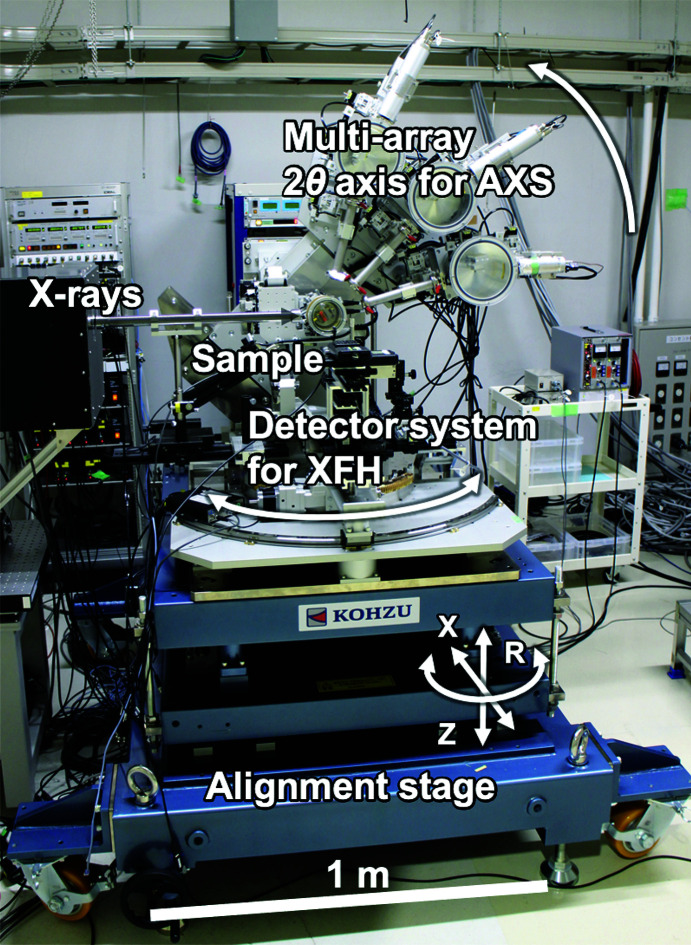
Photograph of the diffractometer for XFH and AXS on the BL47XU beamline at SPring-8. The dimensions of the instrument are 1.8 m (length) × 1.6 m (width) × 2.2 m (maximum height), with a weight of 2.5 t.

**Figure 2:**
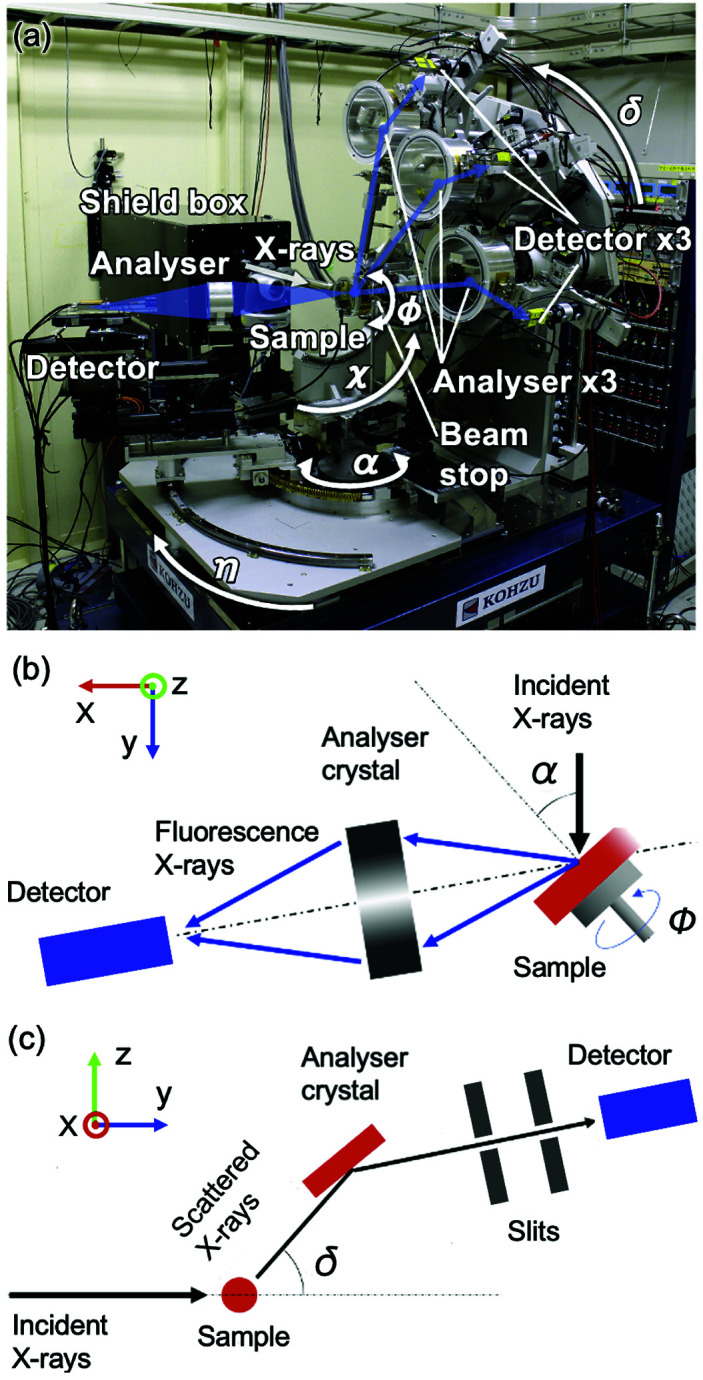
(*a*) Experimental setup for XFH and AXS in the horizontal and vertical scattering planes, respectively. Measurement geometries for (*b*) XFH and (*c*) AXS. The *X* and *Z* directions are the same as those in Fig. 1 ▸.

The diffractometer on the BL47XU beamline at SPring-8 employed in this study is shown in Fig. 1 ▸. The light source of BL47XU is the SPring-8 standard in-vacuum undulator. The incident X-rays in the energy range of 6–37.7 keV are available with a cryogenically cooled Si 111 double-crystal monochromator. A pair of Rh-coated mirrors also can be used to reject higher harmonics and vertically focus the incident beam. The usual 2θ axis (hereafter, δ axis) in the vertical scattering plane was designed for AXS measurements using a crystal analyser to observe a large scattering angle over 120°. A detector system in the horizontal scattering plane was used in the inverse mode for XFH. The combination of an open χ circle with the δ axis was used to determine the crystal orientation of a sample by observing the Bragg reflections without detecting emission lines by a standing wave in a hologram, which is a notable advantage of our diffractometer. In particular, the availability of XFH even to a sample with blurred emission lines is enhanced. All axes mentioned above are on the motorised translation stage (*X* and *Z*) with one manual rotational motion (*R*), which enables the alignment of the instrument to the X-ray beam path within a few tens of micrometres. The incident X-ray optics in the beamline hutch consisted of a precision slit, attenuator, vacuum path, and ion chamber as a beam-flux monitor. A sufficiently low background noise level was achieved by installing a radiation shield box surrounding the incident X-ray optics, adequate lead scattering shields around the detectors, and a beam stop for the incident beam, as shown in Fig. 2 ▸(*a*).

### 2.2. X-ray fluorescence holography

XFH is a technique to observe the atomic resolution hologram produced by interference between fluorescence X-rays from the source atom and those scattered by surrounding atoms. The theoretical foundations of XFH were established by Szöke (1986 ▸), and the first XFH hologram was recorded a decade later by Tegze & Faigel (1996 ▸). In the kinematical approximation, the holographic modulation of the fluorescence yield can be approximated as (Len *et al.*, 1994 ▸) 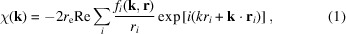
where *r*_e_ and **r**_*i*_ (|**r**_*i*_| = *r*_*i*_) are the classical electron radius and position of the *i*-th scattering atom from the emitter atom of the fluorescence X-rays, respectively; and **k** (|**k**| = *k*) and *f*_*i*_ are the wavevector and atomic form factor of the *i*-th scatterer, respectively. A three-dimensional (3D) atomic image around the emitter atom is reconstructed from the obtained hologram using the Helmholtz–Kirchhoff integral transformation (Barton, 1988 ▸).

XFH has two measurements modes, namely, normal and inverse. Observed intensities using these two modes are essentially the same on the basis of the reciprocity theorem in optics (James, 1962 ▸), that is, if a point source of radiation and an observed point are interchanged, the measured intensity at the new observation point will be the same as that at the previous point. Multiple-energy measurements using the inverse mode by changing the incident X-ray energy have the advantage of reducing the ghost images inherited in XFH (Gog *et al.*, 1996 ▸). Thus, the inverse mode can produce a better reconstructed 3D atomic image than the normal mode. Another advantage is the higher count statistics that can be achieved using the inverse mode.

Therefore, in our instrument, we applied a measurement system in the inverse mode as the standard setup, as shown in Figs. 2 ▸(*a*) and 2 ▸(*b*). Note that the normal mode measurement is also possible using a two-dimensional detector. The XFH detector system in the inverse mode uses three axes for a sample (α, ϕ, and χ) and the η axis for the angle control of the detecting system, composed of an analyser and detector in the horizontal plane. In the inverse mode, we measured the fluorescence yield by changing the angle of the incident X-rays using the α and ϕ axes. A brushless motor was applied for the ϕ axis to achieve continuous rotation in the same rotational direction, which is preferable to effectively measure a hologram and achieve angular precision by XFH. Three translational motions were used to adjust the irradiated area of the sample. For the analyser crystal, we apply a graphite cylindrical analyser (Hayashi *et al.*, 2001 ▸), toroidally bent analyser (Sekioka *et al.*, 2005 ▸), and C-shaped analyser (Happo *et al.*, 2018 ▸, 2024 ▸) for the effective collection of a large solid angle of fluorescence X-rays. Typically, an avalanche photodiode or a solid-state detector is used to collect fluorescence X-ray signals.

### 2.3. Anomalous X-ray scattering

AXS is a method for obtaining element-specific information from X-ray total scatterings (XTSs) using the anomalous term in the complex atomic form factor, *f*, of each element, 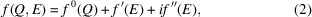
where *Q* is defined by the wavelength of the incident X-rays, λ, and scattering angle, 2θ, as *Q* = (4π/λ)sinθ; *f*′ and *f*′′ are the real and imaginary parts of the energy (*E*)-dependent term, respectively.

In XTS, the pair distribution function, *g*(*r*), is expressed by the total structure factor, *S*(*Q*) (Waseda, 2002 ▸; Egami & Billinge, 2003 ▸): 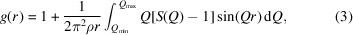
where *r*, ρ, *Q*_min_, and *Q*_max_ represent the distance in real space, atomic number density, and minimum and maximum observed *Q*, respectively. *Q*_max_ determines the real space resolution of the analysis. *S*(*Q*) is experimentally extracted from the XTS intensities, *I*(*Q*), by normalising with *f*^2^ of the constituting elements, while *S*(*Q*) consists of the Faber–Ziman partial structure factors, *S*_*ij*_(*Q*), of the *i*-th and *j*-th elements (Faber & Ziman, 1965 ▸).

The intensity difference, Δ*I*, is derived from normalised scattering intensities measured with two different X-ray energies, *E*_near_ and *E*_far_: 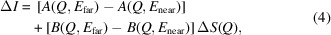
where *A* = 〈*f*^2^〉 − 〈*f*〉^2^ and *B* = 〈*f*〉^2^ are described using chemical averages of the atomic form factors, 〈*f*〉, and squared averages, 〈*f*^2^〉. *E*_near_ and *E*_far_ are close to the absorption edge of a specific element, which are typically at an energy below 30 eV and 300 eV from the edge, respectively. As only *f*′ changes drastically, while *f*′′ remains almost constant in this energy range, this condition yields a simple interpretation of the obtained AXS spectrum. The differential structure factor, Δ*S*(*Q*), is given as a linear combination of *S*_*ij*_(*Q*), *i.e.* Δ*S*(*Q*) = 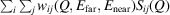, with weighting factors, *w*_*ij*_, according to *f*_*i*_ and *f*_*j*_ (Waseda, 2002 ▸; Egami & Billinge, 2003 ▸). We can define Δ*g*(*r*) by substituting *S*(*Q*) into Δ*S*(*Q*) in equation (3). Δ*S*(*Q*) strongly enhances the related partial structure factors of the *i*-th element and suppresses those of the others (Kohara *et al.*, 2013 ▸), facilitating the element-specific analysis.

Three multi-array detector systems with three crystal analysers arranged every 30° can accelerate high-throughput measurements, as shown in Fig. 2 ▸(*a*). Each AXS detector system comprises slits for incoming and outgoing X-rays towards a crystal analyser, where the double-slit configuration was applied for the outgoing beam, *i.e.* in front of a detector to reduce background noise [Fig. 2 ▸(*c*)]. The analyser crystals were set under vacuum in cylindrical chambers with two X-ray windows made of polyimide films to avoid analyser contamination. The analyser crystal and detector were set to the θ–2θ configuration to measure symmetric reflections from the analyser. The analyser axis was motorised with high precision (0.001° per pulse), which is an order of magnitude finer than those for the other axes. The angle of the 2θ arm can be adjusted to detect the scattered signal with near- and far-energy configurations without readjusting the arm angle by selecting adequate slit sizes. Currently, we have two detector options, namely, plastic scintillation counters and avalanche photodiodes, depending on the energy of the incident X-rays. According to the absorption corrections in the three detectors, *i.e.* asymmetric transmission geometry (Egami & Billinge, 2003 ▸; Rowles & Buckley, 2017 ▸), we recommend the transmission geometry for samples as a standard for a simpler interpretation of absorption corrections while offering both reflection and transmission geometries.

### 2.4. Sample preparation

A natural zeolite (Junnar, Pune District, Maharashtra, India) purchased from N’s Mineral Co. Ltd was used as the scolecite crystal in this study. For the measurements, the crystal was cut and polished to approximately 3 mm × 8 mm × 1 mm. A crystal structure analysis of the sample was performed using a laboratory X-ray source with an Mo target before the synchrotron XFH experiments to clarify the crystal quality. The sharp X-ray diffraction (XRD) peaks revealed that the sample is a fine single crystal of the space group *Cc* (monoclinic), with the structural parameters of *a* = 6.52520 (10) Å, *b* = 18.9769 (3) Å, *c* = 9.7779(2) Å, and β = 108.8570 (6)°, resulting in an *R* factor of 0.0240. Detailed structural information of the scolecite crystal is provided in the supporting information.

The Ag_2_O–ZnO–B_2_O_3_ systems, which have garnered increasing attention in various fields, including catalysis, where Ag quantum clusters are formed in borate glass (Zheng *et al.*, 2023 ▸), were selected for AXS. The 15Ag_2_O–15ZnO–70B_2_O_3_ glass was prepared using the melt-quenching method. Mixtures of raw materials (ZnO, Ag_2_O, and HBO_2_) according to the target composition were melted in a closed alumina crucible at 1300°C for 90 min, followed by pouring into a brass template for quenching.

## 3. Results

### 3.1. 3D atomic imaging by XFH

**Figure 3:**
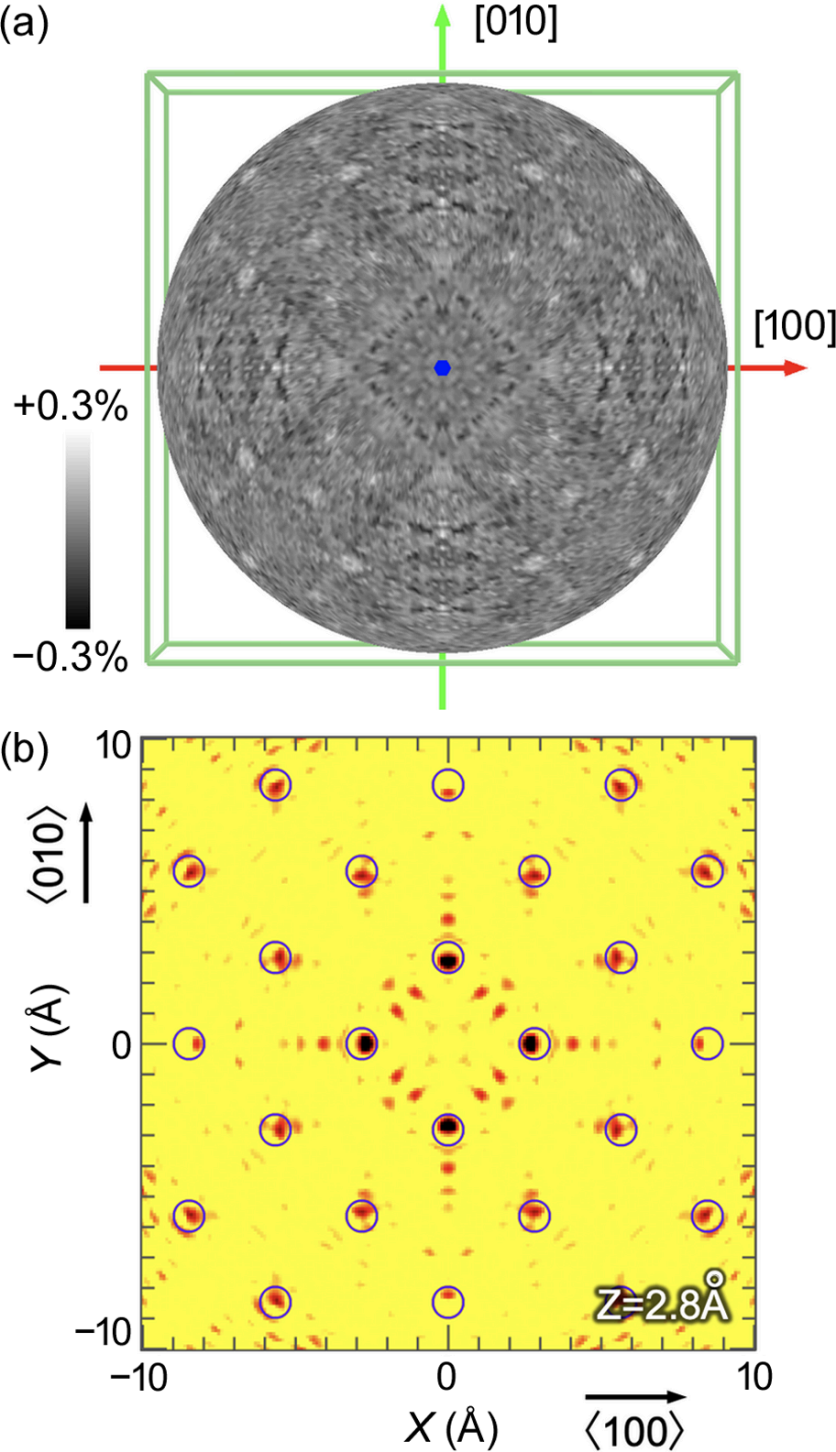
(*a*) Hologram pattern of XFH from a Ge single crystal under 14.5 keV incident X-rays, with intensity variation in the range of ±0.3%. Clear emission lines by a standing wave can be observed. (*b*) Reconstructed Ge atomic image from the hologram. The open circles denote the expected positions of Ge atoms.

Figure 3 ▸(*a*) shows the typical hologram pattern from a Ge single crystal of the Ge *K*α line projected onto the [001] direction measured at 14.5 keV of the incident X-ray energy in the inverse mode. Higher harmonics were rejected using X-ray mirrors. We used a cylindrical graphite crystal analyser (Hayashi *et al.*, 2001 ▸) to collect the fluorescence X-rays. With 50 µm × 50 µm incident X-rays, it took 3 h to complete one hologram. We reconstructed the 3D atomic images using eight holograms measured at the X-ray energies of 11.5–15.0 keV at incremental steps of 0.5 keV. Each hologram was extracted from the measured data using symmetry operations based on the crystal orientation, as determined from the emission lines caused by a standing wave.

Figure 3 ▸(*b*) shows the *X*–*Y*-reconstructed image of the section at *z* = 2.8 Å from the Ge atom as the source point, where the *X* and *Y* axes correspond to the 〈100〉 and 〈010〉 directions, respectively. The reconstructed image from the holograms shows the surrounding Ge atoms at atomic resolution, as expected, towards the central Ge atom as the source of fluorescence X-rays. We used the *3D-AIR-IMAGE* software for the data analysis and visualisation of the atoms (Matsushita, 2015 ▸; Matsushita *et al.*, 2018 ▸).

Besides, we present the XFH results of the scolecite as an example that has a hologram with blurred emission lines caused by a standing wave, posing difficulties in refining the crystal orientation of the sample. Symmetry operations on the observed hologram based on the crystal orientation are required to produce a hologram covering a wide solid angle because observing the complete hologram over the entire solid angle is experimentally limited, even in inverse mode. Therefore, knowing the fine crystal orientation at an angle of less than 0.1° is essential to obtain a precise reconstructed image. Determining the crystal orientation by measuring the Bragg reflections through single-crystal XRD can address this difficulty. Our instrument unifying the XFH and AXS configurations enables us to employ this approach.

**Figure 4:**
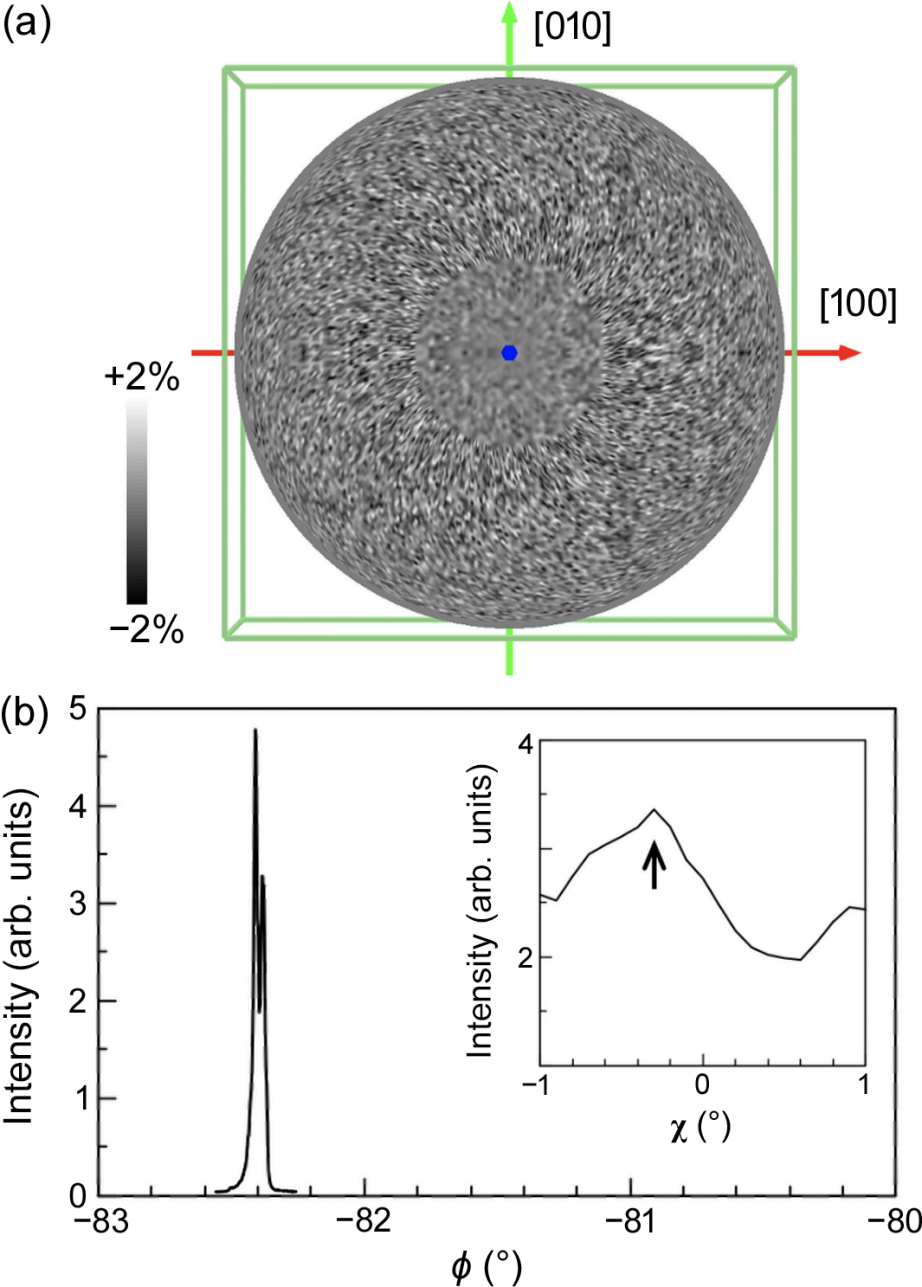
(*a*) Hologram pattern of XFH from a scolecite single crystal, with extremely blurred emission lines. (*b*) RC of the 001 Bragg reflection from the scolecite. The inset shows χ scan results for the angle refinement.

Figure 4 ▸(*a*) shows the hologram from the scolecite crystal of the Ca *K*α line projected onto the [001] direction with 9.2 keV incident X-rays in the inverse mode. Eight holograms with a beam size of less than 50 µm in the X-ray energy range of 9.2–13.2 keV measured in steps of 0.5 keV were used for the analysis. Figure 4 ▸(*b*) shows the rocking curve (RC) of the 001 Bragg reflection from the scolecite. From the angular set (χ, ϕ, α, and δ) obtained from the fine Bragg peak, we successfully obtained the crystal orientation of the scolecite crystal and executed symmetry operations on the hologram. The angular resolution was sufficient for determining the crystal orientation though the Bragg peak split in the ϕ scan.

**Figure 5:**
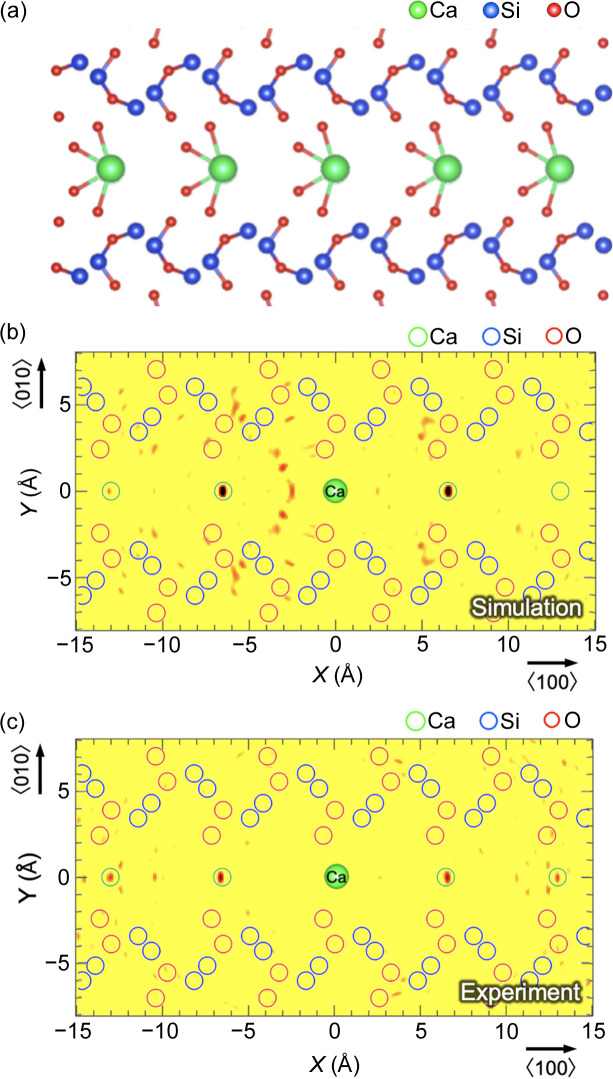
(*a*) Crystal structure of scolecite drawn using *VESTA* (Momma & Izumi, 2011 ▸). Reconstructed images of scolecite obtained by (*b*) simulation and (*c*) experiment. Ca atoms were clearly visualised from the experiment, indicated as green open circles.

As expected from the crystal structure of the scolecite shown in Fig. 5 ▸(*a*), atomic images corresponding to the surrounding Ca atoms are clearly observed, as indicated by the green open circles in the reconstructed image, as shown in Fig. 5 ▸(*c*). These results are in good agreement with our simulation results [Fig. 5 ▸(*b*)], confirming the ability of our system to visualise local atoms with a hologram, even with blurred emission lines by a standing wave.

### 3.2. Element-specific analysis by AXS

**Figure 6:**
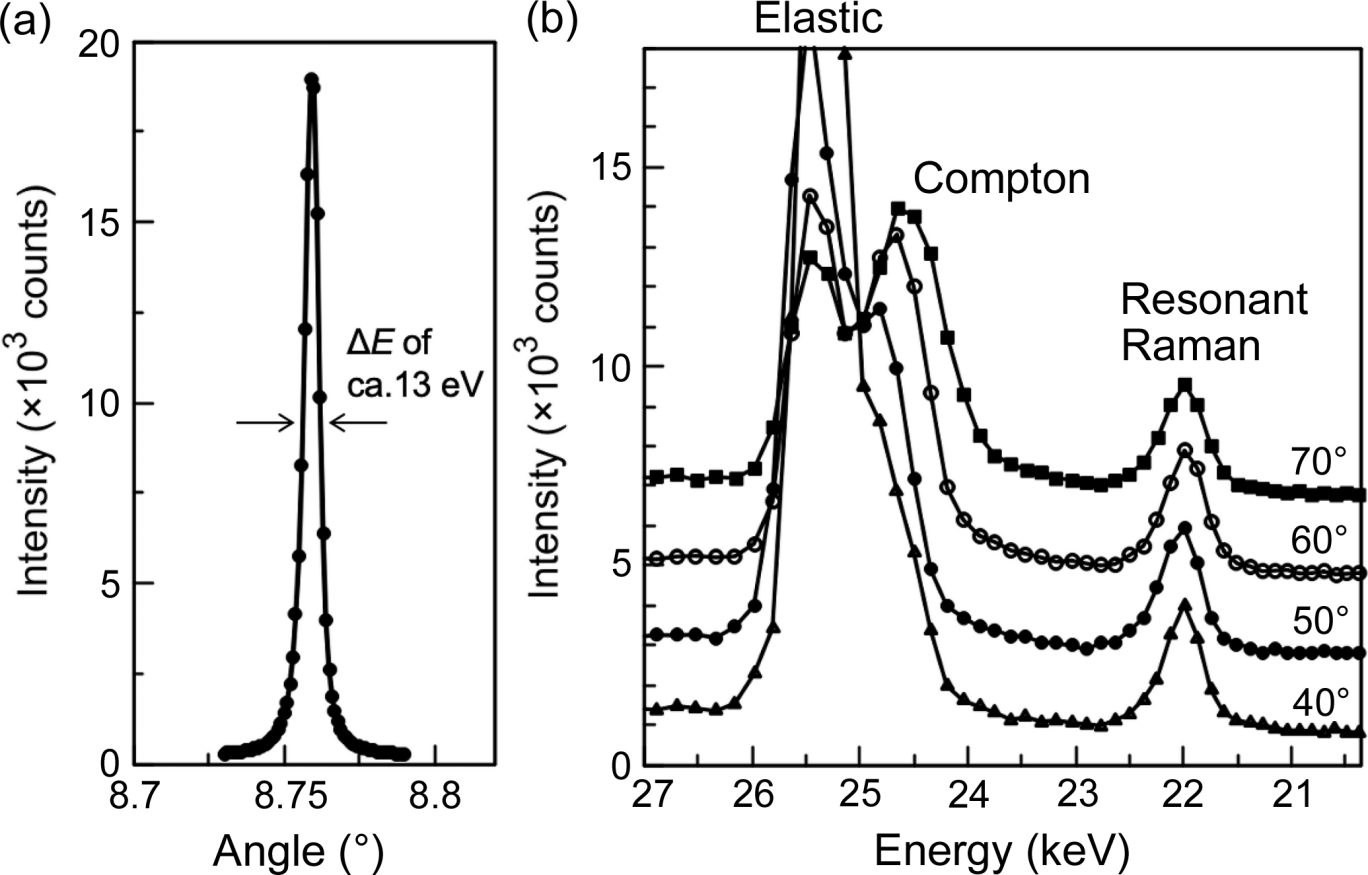
(*a*) RC of the 002 reflection from an LiF analyser crystal with 20 keV X-rays, of which the angular width corresponds to the energy resolution (Δ*E*) of approximately 13 eV. (*b*) Energy spectrum using the LiF analyser in a coarse resolution mode with different scattering angles (40°–70°). Spectra for higher angles are displayed upward for clarity. Elastic and Compton components are distinctly resolved in addition to resonant Raman scattering.

The RC of the LiF analyser crystal (OKEN Co. Ltd) is shown in Fig. 6 ▸(*a*) for the 002 Bragg reflection with an incident X-ray energy of 20 keV. The full width at half-maximum (FWHM), Δθ, of the RC was 0.0059° (*ca* 13 eV in Δ*E*), corresponding to less than 0.1% Δ*E*/*E*, which is sufficient to resolve elastic, Compton, resonant Raman scattering, and fluorescence observed in X-ray scattering (Fischer *et al.*, 2006 ▸). The energy resolution is defined by Δ*E*/*E* = Δθcotθ_B_, where θ_B_ denotes the Bragg angle. The observed energy spectrum of the 15Ag_2_O–15ZnO–70B_2_O_3_ glass at an X-ray energy (25.484 keV) close to the Ag absorption edge obtained by angular scanning of the analyser is shown in Fig. 6 ▸(*b*). As expected, the Compton component increased and gradually shifted from the elastic components in terms of energy according to the scattering angle. The resonant Raman components are independent of the scattering angle. These results highlight the suitability of the LiF crystal for reducing the unwelcome background, including Compton and resonant Raman scatterings, to an AXS spectrum, while maintaining an acceptable energy window for elastic scattering.

**Figure 7:**
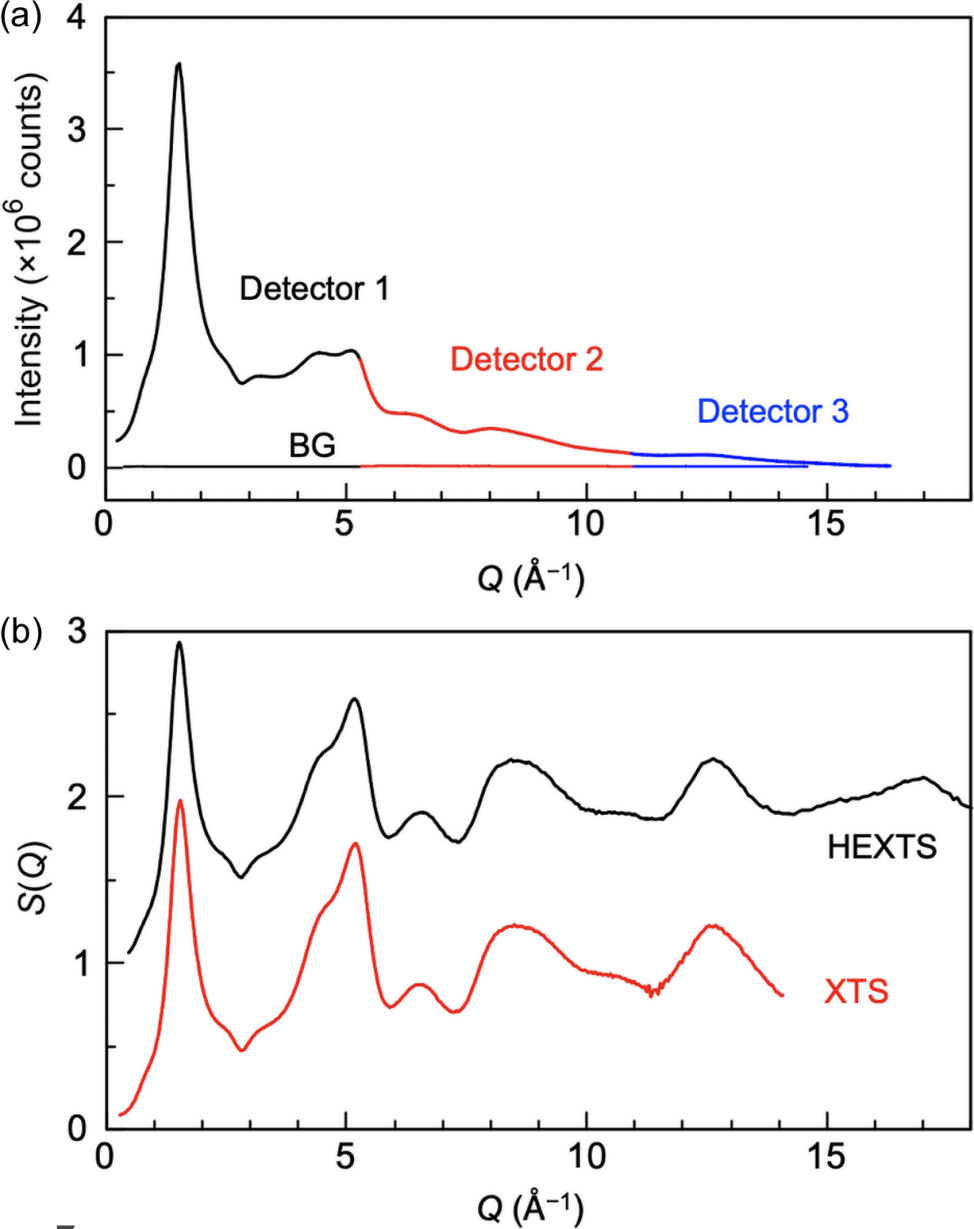
(*a*) XTS of silica glass using the three-detector array system with 20 keV X-rays in 1 h (accumulation time of 20 s in each point). Instrument backgrounds (BGs) are plotted together. (*b*) Total structure factor, *S*(*Q*), of silica glass derived from the data in (*a*). For comparison, HEXTS data are displayed upward by 1 for clarity.

Using the LiF analyser crystal configurations, we measured the XTS of silica glass with three detector arrays at an X-ray energy of 20 keV. The transmission geometry was applied to a 10 mm × 10 mm × 1 mm silica glass plate. Figure 7 ▸(*a*) represents the XTS from the silica sample as the standard sample with a measurement time of 1 h. The scattering data obtained by the three detectors with the analysers depicted good agreement. For this data connection, we used an overlapping *Q* region to calibrate the data. Notably, sufficient count statistics required for AXS were achieved because the total count at the first sharp diffraction peak reached over 3.5 × 10^6^ (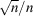 is less than 0.1%). Therefore, our instrument achieved high-throughput measurements three times faster than conventional instruments with a single detector.

The systematic error owing to the acceptance angle instability of the analyser crystal in a wide-range scan, resulting from the instrument precision and misalignment, was reduced considerably by limiting the scan range from 0° to 30° in each system. *S*(*Q*) was derived using corrections for self-absorption, normalisation by the flux of incident X-rays, and combining the datasets from the three detector systems. As shown in Fig. 7 ▸(*b*), the measured *S*(*Q*) using our instrument and the reference *S*(*Q*) observed by high-energy (HE) XTS (Ohara *et al.*, 2021 ▸) are in excellent agreement with sufficient statistics, even at *Q* over 10 Å^−1^.

**Figure 8:**
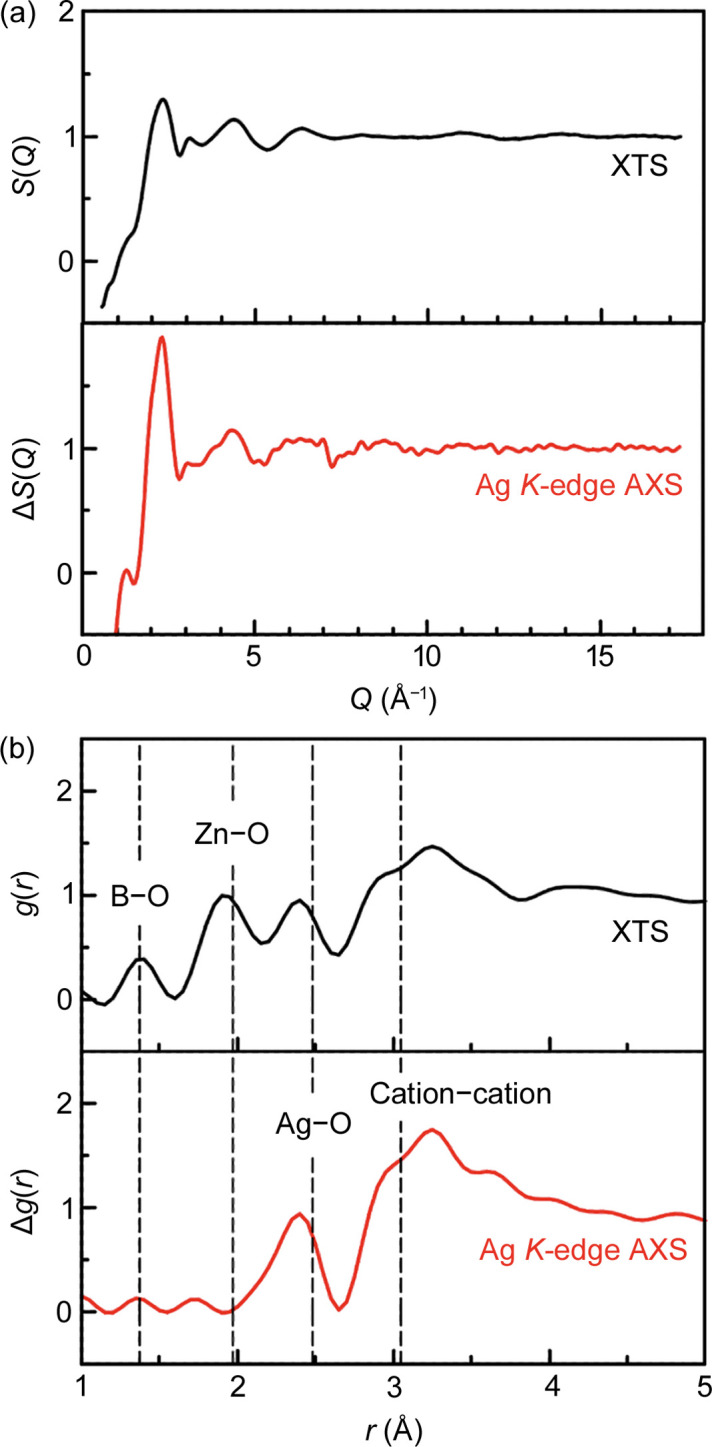
(*a*) Total structure factor, *S*(*Q*), and differential structure factor, Δ*S*(*Q*), obtained from XTS and AXS, respectively, of the 15Ag_2_O–15ZnO–70B_2_O_3_ glass. (*b*) Total *g*(*r*) and Δ*g*(*r*) derived from the data in (*a*). The total *g*(*r*) shows peaks corresponding to B—O, Zn—O, Ag—O, and cation—cation correlations, whereas Δ*g*(*r*) highly enhanced the Ag—O bond length, depicting element-specific features. Dashed lines are provided as guides.

Finally, we present the typical AXS data obtained for the 15Ag_2_O–15ZnO–70B_2_O_3_ glass. Figure 8 ▸(*a*) presents Δ*S*(*Q*), the differential structure factor between the two *S*(*Q*) measured with X-rays near to (−30 eV) and far from (−300 eV) Ag *K* absorption edge (25.514 keV), together with *S*(*Q*). Δ*S*(*Q*) possesses Ag-specific structural information owing to the difference in the anomalous term (*f*′) of the Ag form factor. The total *g*(*r*) and Δ*g*(*r*) were derived from the Fourier transform of *S*(*Q*) and Δ*S*(*Q*), respectively, with *Q*_max_ = 17.3 Å^−1^, as shown in Fig. 8 ▸(*b*). Bond lengths of the B_2_O_3_–Ag_2_O glass extracted by neutron scattering (Ushida *et al.*, 2001 ▸) are also indicated by dashed lines, together with those of the MoO_3_–ZnO–B_2_O_3_ glass confirmed by neutron scattering and reverse Monte Carlo modelling (Fabian *et al.*, 2016 ▸). Evidently, Δ*g*(*r*) distinctly enhanced the peak corresponding to the Ag—O correlation by the element-specific feature, whereas total *g*(*r*) has a less visible peak of Ag—O together with those of B—O and Zn—O bond lengths.

## 4. Discussion

In Section 3, we presented typical XFH and AXS data using our instrument. The unification of the XFH and AXS configurations into one instrument was beneficial because both methods offered element-specific analyses using wavelength tunability of synchrotron X-rays and required photon-hungry experiments that need count statistics finer than 0.1%, showing similarities. XFH could obtain deeper insights into the structures of local disorders at the atomic level in a crystal, *e.g.* defects, substitutions, and distortions. Meanwhile, AXS provided information on ordering beyond the nearest-neighbour distance in disordered materials, for example, both short- and intermediate-range order in glass, representative of disordered materials. In other words, our diffractometer offers dual-experimental approaches to reveal local structures in both material states (*i.e.* crystal and amorphous phases), which play pivotal roles in the emergence of functionalities. This will lead to unified understandings of the functionalities across these contrasting states from the structural viewpoints.

In Section 3.1, we demonstrated that our system can image local atoms by XFH using scolecite as an example, which had blurred emission lines by a standing wave in a hologram. Owing to another degree of freedom (an open χ circle) and the δ axis used in AXS, our instrument can act as the four-axis diffractometer that enables determining a crystal orientation required for symmetry operations on the observed hologram. This is the great advantage for XFH measurements using our instrument, which unifies XFH and AXS configurations, increasing tractable targets.

In Section 3.2, we discussed the results of using an LiF crystal with 002 reflection as the standard analyser of *ca* 0.1% bandwidth for accurate subtraction of inelastic and re-emission components to extract the elastic scattering in AXS. In such a narrow-bandwidth analyser, fine angular alignment of the crystal to the scattering plane was required to obtain reliable data. As three detectors with analysers were arranged every 30° in our system, each scan range was limited to 0–30° narrower than in a conventional one-detector system, reducing the systematic errors caused by the acceptance angles of the analyser crystals. In addition to the high throughput, this is another advantage of the multi-array detector systems. An analyser crystal adequate for the experimental demand can be selected. Graphite, which exhibits wider FWHMs in RC than LiF, is a possible solution for a dilute system that requires higher flux for count statistics.

The large working distance design results in the availability of a microbeam option using refractive lenses (typically in the X-ray energy range of 6–25 keV) for microscopic measurements. The goniometer for a sample that includes three axes is also interchangeable with, for example, a sample preparation instrument for *in situ* observation.

## 5. Conclusions

We developed a carry-in diffractometer in SPring-8 that utilises both XFH and AXS, realising element-specific analyses with atomic resolution using the wavelength tunability of the synchrotron X-ray source. The combination of XFH and AXS configurations facilitates the determination of crystal orientation via diffractometry. This feature enables the application of XFH even for crystals with blurred emission lines caused by a standing wave in a hologram. Moreover, the three multi-array detector systems with three crystal analysers, which offer sufficient energy resolutions to resolve elastic, Compton, resonant Raman scattering, and fluorescence, realise high-throughput measurements with sufficient count statistics required for AXS. These features enable us to tackle disorder in crystals and short- and intermediate-range order in amorphous materials such as glass, and increase tractable targets by XFH and AXS, which have novel functionalities.

## 6. Related literature

The following references, not cited in the main body of the paper, have been cited in the supporting information: Fälth & Hansen (1979 ▸); Palatinus & Chapuis (2007 ▸); Petříček *et al.* (2014 ▸).

## Supplementary Material

On crystal structure determination of scolecite: Fig. S1 and Tables S1 and S2. DOI: 10.1107/S1600577524011366/ok5128sup1.pdf

Crystal structure: contains datablock(s) global, I. DOI: 10.1107/S1600577524011366/ok5128sup2.cif
