# Supplementary material for: Diffractometer for element-specific analysis on local structures using a combination of X-ray fluorescence holography and anomalous X-ray scattering
Source: J Synchrotron Radiat. 2025 Jan 1;32(Pt 1):125–32. doi: 10.1107/S1600577524011366 (PMC11708849; doi:10.1107/S1600577524011366)
Supplement: Supplementary file 1 [file s-32-00125-sup1.pdf]

Supporting information:

Diffractometer for element specific analysis on local structures in combination with X-ray fluorescence holography and anomalous X-ray scattering

Hiroo Tajiri [a*], Shinji Kohara [b,a], Koji Kimura [c,a,b], Sekhar Halubai [c,a], Haruto Morimoto [c], Naohisa Happo [d,a], Jens R. Stellhorn [e], Yohei Onodera [b,f], Xvsheng Qiao [g],Daisuke Urushi-hara [h], Peidong Hu [i], Toru Wakihara [i], Toyohiko Kinoshita [a] and Koichi Hayashi [c,a]

[a] Japan Synchrotron Radiation Research Institute, Hyogo, 679-5198, Japan, [b] Center for Basic Research on Materials, National Institute for Materials Science, Ibaraki, 305-0047, Japan, [c] Department of Physical Science and Engineering, Nagoya Institute of technology, Nagoya, 466-8555, Japan, [d] Graduate School of Information Sciences, Hiroshima City University, Hiroshima, 731-3194, Japan, [e] Institute of Advanced Mate- rials Research and Development, Shimane University, Matsue, 690-8504, Japan, [f] Institute for Integrated Radiation and Nuclear Science, Kyoto University, Osaka, 590-0494, Japan, [g] State Key Laboratory of Sil- icon Materials & School of Materials Science and Engineering, Zhejiang University, Hangzhou, 310027, China, [h] Division of Advanced Ceramics, Nagoya Institute of technology, Nagoya, 466-8555, Japan, and [i] Institute of Engineering Innovation, School of Engineering, The University of Tokyo, Tokyo, 113-8656, Japan. Correspondence e-mail: **tajiri@spring8.or.jp**

1. Crystal structure determination of scolecite

Diffraction data were collected using a single-crystal X-ray diffractometer (D8 VENTURE, Bruker) equipped with an Mo *K*α X-ray source (50 kV, 1 mA). A single crystal of dimensions of approximately 80×60×40 μm was mounted on a borosilicate glass needle using glue. The initial structure model was calculated using the Superflip program based on the charge-flipping algorithm [1]. Crystal structure analysis was conducted using the JANA2006 program package [2], and the analyzed crystal structure was visualized using the VESTA program [3].

Single-crystal XRD data obtained for scolecite were indexed to a monoclinic unit cell with space group *Cc*. The unit cell dimensions were a=6.52520(10) Å, b=18.9769(3) Å, c=9.7779(2) Å, and β=108.8570(6)°. For the actual chemical composition of scolecite, $CaAl_2Si_3O_{10}{\cdot}3H_2O$, we adopted $CaSi_5O_{10}{\cdot}3O$ as an approximate model to find the initial structure model. We regarded Al (Z=13), having very close numbers of electrons to Si, as identical to Si (Z=14), and omitted H ions due to their small scattering factor, too. The initial structure model obtained by the charge-flipping method represents the 19 independent sites in the unit cell. Then, we refined all coordination sites and atomic

displacement parameters (*U*) based on the actual chemical composition including Al and water. The reliability indices were R = 2.40% and wR = 7.30%. The Flack parameter was 0.01(2), which represents a monodomain crystal concerning the inversion twin. The crystal structure model refined in this experiment is the same as reported in previous studies [4]. The refined crystal structure model is shown in Figure S1, and the crystal data and structural parameters are summarized in Tables S1 and S2, respectively. The CIF is also available as supporting information.

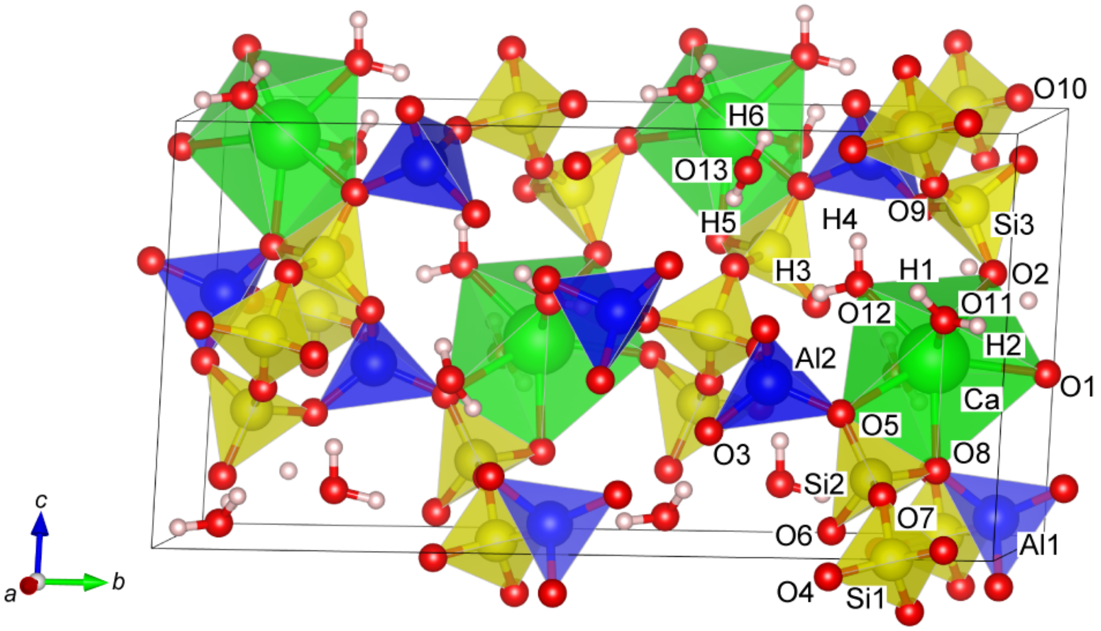

Figure S1. Crystal structure model of scolecite. Atoms colors: Ca (green), Al (blue), Si (yellow), O (red), and H (pink).

Table S1. Crystal data and experimental conditions of XRD for scolecite.

Chemical formula	$CaAl_2Si_3O_{10}\cdot 3H_2O$
Space group	*Cc* (No. 9)
a / Å	6.52520(10)
b / Å	18.9769(3)
c / Å	9.7779(2)
β / °	108.8570(6)
V / Å^3	1145.79(3)
Z	4
Measured reflection	66889
Unique reflection	4918
R(int)	0.0363
Collection range	$-13 \le h \le 13$
	$-37 \le k \le 37$
	$-19 \le l \le 19$
R [$F^2 > 3\sigma(F^2)$]	0.0240
$wR(F^2)$	0.0730

Table S2. Structural parameters and atomic displacement parameters ($Å^2$) for scolecite.

Site	Wyckoff position	g	x	y	z	U_{iso}^*/U_{eq}
Ca1	4*a*	1	0.10920(9)	0.393259(18)	0.44799(6)	0.01279(9)
Al1	4*a*	1	0.92202(10)	0.46661(3)	0.06623(7)	0.00754(12)
Al2	4*a*	1	0.33439(11)	0.21209(3)	0.40026(7)	0.00771(12)
Si1	4*a*	1	0.5	0.37938(2)	0	0.00788(11)
Si2	4*a*	1	0.20879(10)	0.33259(2)	0.16895(6)	0.00732(11)
Si3	4*a*	1	0.52980(10)	0.08182(2)	0.29989(6)	0.00746(11)
O1	4*a*	1	0.4952(2)	0.02117(7)	0.40923(14)	0.0125(3)
O2	4*a*	1	0.4955(2)	0.04953(6)	0.14160(13)	0.0101(3)
O3	4*a*	1	0.3623(2)	0.14568(7)	0.28522(15)	0.0138(3)
O4	4*a*	1	0.1264(2)	0.18708(7)	0.46554(16)	0.0138(4)
O5	4*a*	1	0.2731(2)	0.29604(7)	0.32592(14)	0.0111(3)
O6	4*a*	1	0.0812(2)	0.28149(7)	0.03937(15)	0.0135(3)
O7	4*a*	1	0.4325(2)	0.36014(7)	0.14335(14)	0.0126(3)
O8	4*a*	1	0.0677(2)	0.40120(7)	0.18449(14)	0.0120(3)
O9	4*a*	1	0.7804(2)	0.10844(7)	0.36576(15)	0.0140(3)
O10	4*a*	1	0.6478(2)	0.44930(7)	0.03223(15)	0.0133(3)
O11	4*a*	1	0.9674(3)	0.07623(10)	0.0598(3)	0.0360(6)
O12	4*a*	1	0.7048(3)	0.19534(9)	0.12548(18)	0.0276(5)
O13	4*a*	1	0.7826(3)	0.33045(12)	0.3917(2)	0.0323(6)
H1	4*a*	1	1.078(4)	0.1030(14)	0.130(3)	0.0432*
H2	4*a*	1	1.036(4)	0.0324(9)	0.052(4)	0.0432*
H3	4*a*	1	0.662(5)	0.2433(5)	0.100(3)	0.0332*
H4	4*a*	1	0.768(6)	0.1958(14)	0.2288(5)	0.0332*
H5	4*a*	1	0.664(3)	0.3523(18)	0.319(3)	0.0388*
H6	4*a*	1	0.722(5)	0.3161(19)	0.465(3)	0.0388*

Table S2. (continued)

Site	U_{11}	U_{22}	U_{33}	U_{12}	U_{13}	U_{23}
Ca	0.01464(13)	0.01219(13)	0.01241(13)	0.00067(11)	0.00558(10)	-0.00066(10)
Si1	0.00812(19)	0.00653(19)	0.00843(19)	0.00009(15)	0.00328(15)	-0.00020(15)
Si2	0.00784(18)	0.00670(19)	0.00862(19)	0.00057(15)	0.00269(15)	0.00064(15)
Si3	0.00678(16)	0.00797(17)	0.00927(17)	0.00032(13)	0.00313(13)	0.00026(13)
Si4	0.00740(17)	0.00697(16)	0.00750(17)	0.00075(13)	0.00226(13)	0.00075(13)
Si5	0.00879(17)	0.00732(17)	0.00642(17)	0.00014(13)	0.00266(13)	-0.00079(13)
O1	0.0192(5)	0.0104(5)	0.0103(5)	0.0010(4)	0.0082(4)	0.0016(4)
O2	0.0141(5)	0.0083(5)	0.0083(4)	-0.0020(4)	0.0043(4)	-0.0011(4)
O3	0.0145(5)	0.0118(5)	0.0144(5)	0.0044(4)	0.0037(4)	-0.0039(4)
O4	0.0143(5)	0.0111(5)	0.0198(6)	-0.0035(4)	0.0107(5)	-0.0008(4)
O5	0.0153(5)	0.0091(5)	0.0091(5)	0.0015(4)	0.0041(4)	0.0023(4)
O6	0.0130(5)	0.0136(5)	0.0113(5)	-0.0008(4)	0.0003(4)	-0.0027(4)
O7	0.0107(5)	0.0180(6)	0.0097(5)	-0.0033(4)	0.0042(4)	0.0005(4)
O8	0.0139(5)	0.0096(5)	0.0129(5)	0.0041(4)	0.0049(4)	0.0018(4)
O9	0.0094(5)	0.0185(6)	0.0115(5)	-0.0027(4)	0.0000(4)	-0.0011(4)
O10	0.0104(5)	0.0111(5)	0.0189(6)	-0.0023(4)	0.0052(4)	-0.0005(4)
O11	0.0176(7)	0.0249(8)	0.0594(13)	0.0058(6)	0.0038(8)	-0.0078(8)
O12	0.0489(11)	0.0147(7)	0.0178(7)	-0.0039(6)	0.0088(7)	0.0011(5)
O13	0.0168(7)	0.0471(11)	0.0279(8)	-0.0086(7)	-0.0001(6)	0.0085(7)

Supplementary References

[1] L. Palatinus and G. SUPERFLIP Chapuis, "a computer program for the solution of crystal structures by charge flipping in arbitrary dimensions.", *J. Appl. Crystallogr.*, **40**, 786-790 (2007).

[2] V. Petricek, M. Dusek, and L. Palatinus, "Crystallographic computing system JANA2006: General features.", *Z. Kristallogr.*, **229**, 345-352 (2014).

[3] K. Momma and F. Izumi, "VESTA 3 for three-dimensional visualization of crystal, volumetric and morphology data.", *J. Appl. Crystallogr.*, **44**, 1272-1276 (2011).

[4] L. Fälth and S. Hansen, "Structure of Scolecite from Poona, India.", *Acta Crystallogr. B*, **35**, 1877-1880 (1979).
